# Impact of cocaine abuse on HIV pathogenesis

**DOI:** 10.3389/fmicb.2015.01111

**Published:** 2015-10-20

**Authors:** Sabyasachi Dash, Muthukumar Balasubramaniam, Fernando Villalta, Chandravanu Dash, Jui Pandhare

**Affiliations:** ^1^Center for AIDS Health Disparities Research, Meharry Medical College, Nashville, TN, USA; ^2^School of Graduate Studies and Research, Meharry Medical College, Nashville, TN, USA; ^3^Department of Biochemistry and Cancer Biology, Meharry Medical College, Nashville, TN, USA; ^4^Department of Microbiology and Immunology, Meharry Medical College, Nashville, TN, USA

**Keywords:** drug use, cocaine, HIV, AIDS, pathogenesis

## Abstract

Over 1.2 million people in the United States are infected with the human immunodeficiency virus type 1 (HIV-1). Tremendous progress has been made over the past three decades on many fronts in the prevention and treatment of HIV-1 disease. However, HIV-1 infection is incurable and antiretroviral drugs continue to remain the only effective treatment option for HIV infected patients. Unfortunately, only three out of ten HIV-1 infected individuals in the US have the virus under control. Thus, majority of HIV-1 infected individuals in the US are either unaware of their infection status or not connected/retained to care or are non-adherent to antiretroviral therapy (ART). This national public health crisis, as well as the ongoing global HIV/AIDS pandemic, is further exacerbated by substance abuse, which serves as a powerful cofactor at every stage of HIV/AIDS including transmission, diagnosis, pathogenesis, and treatment. Clinical studies indicate that substance abuse may increase viral load, accelerate disease progression and worsen AIDS-related mortality even among ART-adherent patients. However, confirming a direct causal link between substance abuse and HIV/AIDS in human patients remains a highly challenging endeavor. In this review we will discuss the recent and past developments in clinical and basic science research on the effects of cocaine abuse on HIV-1 pathogenesis.

## Introduction

The ongoing HIV/AIDS pandemic has claimed around 39 million human lives, and over 35 million individuals are currently living with HIV-1^1^ infection (Centers for Disease Control and Prevention, 2012). HIV/AIDS research, despite making tremendous strides in the last three decades, is yet to yield a preventative vaccine. Antiretroviral therapy (ART) continues to remain the sole treatment option and the current standard of care for HIV-infected individuals (Walensky et al., 2006). ART has dramatically reduced HIV/AIDS-related mortality and has been highly effective in controlling the virus in ART-adherent patients (Walensky et al., 2006). Recent studies also suggest that ART prophylaxis may help reduce transmission of HIV-1 among high-risk groups (Grant et al., 2010; Cohen et al., 2011; Marrazzo et al., 2015; Siemieniuk and Bogoch, 2015). Despite such advances in managing the HIV/AIDS disease, latest report from the Centers of Disease Control and Prevention (CDC) indicate that over two thirds of the 1.2 million living with HIV-1 infection in the US do not have their virus under control (Centers for Disease Control and Prevention, 2012). Perhaps, a more troubling statistic is that, around 20% of those individuals are unaware of their status (Centers for Disease Control and Prevention, 2012), which also highlights the challenges in controlling the HIV epidemic even in a resource rich setting. The HIV/AIDS crisis is further exacerbated by substance abuse that serves as a powerful cofactor at every stage of HIV epidemic including transmission, diagnosis, pathogenesis, and adherence to therapy (Friedman et al., 2006; Sohler et al., 2007; Cofrancesco et al., 2008; Kipp et al., 2011). Clinical studies implicate substance abuse in increased viral load, accelerated disease progression and worsening of AIDS-related mortality—even among ART-adherent patients (Anthony et al., 1991; Arnsten et al., 2001, 2002; Friedman et al., 2006; Sohler et al., 2007; Cofrancesco et al., 2008; Kipp et al., 2011). Therefore, substance abuse continues to be a major obstacle in combating this global pandemic. It is increasingly becoming evident that drugs such as methamphetamine, cocaine, opiates, marijuana, and others regulate HIV-1 infection/replication *in vitro* and in animal models (Wang and Ho, 2011). Furthermore, new research is shedding light on the negative effects of substance abuse on immune system that controls viral infections (Wang and Ho, 2011). Overall, these studies are paving ways to better understand the comorbid condition of drug use and HIV/AIDS. In this review we will focus on the effects of cocaine abuse on HIV-1 pathogenesis, with a special emphasis on the implications of the interplay between cocaine and the virus in the brain.

### Cocaine Abuse and Mechanism of Action

Cocaine, a commonly used illicit drug, is an alkaloid derived from the leaves of the coca plant *Erythroxylum coca*. According to the National Survey on Drug Use and Health (NSDUH), in 2013, around 1.5 million Americans aged 12 and older had abused cocaine in some form^2^. Cocaine is a potent vasoconstrictor and a powerful stimulant of the brain, and abuse of this compound leads to severe medical complications including neurological effects (headaches, fainting attacks, hemorrhagic brain strokes, central nervous system (CNS) vasculitis and encephalopathies), cardiovascular effects (cardiac arrhythmia and heart attacks), and gastrointestinal complications (Daras, 1996; Riezzo et al., 2012; Marasco et al., 2014). Delirium and seizures significantly contribute to the cocaine-induced morbidity and mortality, while movement disorders and cognitive motor dysfunction are also observed in long-term cocaine users (Nnadi et al., 2005). Cocaine primarily enhances the dopamine neurotransmission in the brain, thereby causing amplification and reactivity of sensory cues (Venton et al., 2006; Little et al., 2009). Cocaine directly elevates the synaptic dopamine levels in the mesocorticolimbic system by binding to the presynaptic dopamine transporters (DATs) and inhibiting dopamine reuptake (Ritz et al., 1987; Kalivas and Volkow, 2005; Marasco et al., 2014). This inhibition is considered to be the main contributor to the reinforcement and behavioral properties of cocaine. The elevated dopamine levels in the synaptic cleft result in increased activation of type 1 and type 2 dopamine receptors, while the inhibition of dopamine reuptake significantly contributes to the reinforcement and behavioral properties of cocaine (Anderson and Pierce, 2005; Schmidt and Pierce, 2006; Graham et al., 2007). While acute cocaine exposure promotes dopamine activity, chronic cocaine abuse has been shown to deregulate striatal dopamine signaling (Willuhn et al., 2014). Cocaine is also known to block the reuptake of other monoamine neurotransmitters including norepinephrine and serotonin, and also impact other excitatory neurotransmitters like glutamate and its receptors (Howell and Cunningham, 2015).

### Effects of Cocaine Abuse on HIV pathogenesis

Substance abuse is associated with increased HIV transmission, delayed diagnosis, delayed initiation of therapy, and poor adherence to therapy, which all constitute important barriers for combating the HIV pandemic (Khalsa and Royal, 2004; Cabral, 2006; Friedman et al., 2006; Kipp et al., 2011). Substance abuse also negatively affects the management of HIV/AIDS as roughly one-third of HIV/AIDS patients are intravenous drug users in the United States (Donahoe, 1990). Several studies have reported associations between drug use and HIV-associated clinical outcomes, including the Women’s Interagency HIV study that found substance abuse to be an independent predictor of progression to AIDS and AIDS-related mortality (Cook et al., 2008), and the report by Baum et al., suggesting that substance abuse accelerates HIV disease progression independent of adherence to ART (Baum et al., 2009). Along with clinical evidence, experimental studies have shown that illicit drugs such as amphetamine, marijuana, opiates, nicotine, and cocaine are associated with immunomodulation and the pathogenesis of AIDS (Friedman et al., 2003; Pandhare and Dash, 2011). These drugs cause immunosuppression, modulate the expression of chemokine receptors on CD4^+^ T lymphocytes, and influence the outcome of HIV-1 infection (Battjes et al., 1988; Donahoe and Falek, 1988; Nair et al., 2000). It is important to point out that there are also several studies that could not find an association between substance use and HIV/AIDS (Junghans et al., 1999; Pezzotti et al., 1999; Chao et al., 2008). Nevertheless, the high prevalence of substance abuse among HIV-1 infected individuals (Pence et al., 2008) and the lack of molecular studies warrant further research to decipher the effects of substance abuse on HIV pathogenesis.

#### Cocaine Abuse Accentuates HIV-1 Pathogenesis

Cocaine is a commonly abused drug among HIV-1 infected individuals (Pence et al., 2008) and several studies suggest a link between cocaine use and worsening of HIV disease (Chaisson et al., 1989; Anthony et al., 1991; Siddiqui et al., 1993; Larrat et al., 1996; Cofrancesco et al., 2008; Cook et al., 2008; Baum et al., 2009; Cook, 2011). There is evidence that cocaine use results in lack of virologic suppression and accelerated decline of CD4^+^ T cells even among ART-adherent patients (Carrico et al., 2008; Cofrancesco et al., 2008; Cook et al., 2008; Cook, 2011; Rasbach et al., 2013). For instance, frequent cocaine users exhibit increased risk of worsening HIV-1 disease progression (Webber et al., 1999; Vittinghoff et al., 2001), active cocaine and alcohol use is a strong predictor of failure to maintain viral suppression (Webber et al., 1999; Arnsten et al., 2001, 2002), and cocaine use may result in inferior virologic and immunologic responses to ART (Lucas et al., 2001, 2002, 2006). However, as mentioned previously, some studies had found no significant association between cocaine abuse and HIV-1 disease progression (Pezzotti et al., 1999; Chao et al., 2008). One potential explanation for this discrepancy could be that the effects of cocaine on disease progression may depend in part to non-adherence to ART, as substance abuse is often associated with reduced adherence and/or access to ART (Hinkin et al., 2007). However, it does not appear to be the case as evidenced from several studies, wherein, even when controlled for ART, cocaine users showed higher viral load and were twice as likely to progress to AIDS (Baum et al., 2009), were found less likely to suppress their viral load while taking ART (Palepu et al., 2003), and active cocaine use was associated with lack of virologic suppression independent of ART adherence (Rasbach et al., 2013). Although the mechanism remains largely unclear, increased viral replication and accelerated CD4^+^ T cell decline has been suggested to play key roles for the accelerated disease seen in cocaine abusing HIV-1 patients.

#### Cocaine Enhances HIV-1 Replication

*In vivo* studies on the interactions between cocaine and HIV-1 under physiologic conditions have been conducted in a human-mouse hybrid model (huPBL-SCID mouse; Roth et al., 2002, 2005; Kim et al., 2015). In this model, cocaine administration led to enhanced HIV-1 infection and a significant rise in circulating virus load (Roth et al., 2002, 2005; Kim et al., 2015). Several *in vitro* studies have also demonstrated the potentiating effects of cocaine on HIV-1 infection/replication in monocyte derived macrophages (MDMs; Dhillon et al., 2007), dendritic cells (Napuri et al., 2013), astrocytes (Reynolds et al., 2006), and peripheral blood mononuclear cells (PBMCs) (Peterson et al., 1991, 1992; Bagasra and Pomerantz, 1993). We and others have shown that cocaine also promotes HIV-1 infection/replication in CD4^+^ T cells (Mantri et al., 2012; Kim et al., 2013; Addai et al., 2015). Further, compared to non-drug users, cells from cocaine addicts readily supported HIV-1 replication and AIDS-related opportunistic infections in contrast to cells from non-users (Baldwin et al., 1998). Studies have reported that immunomodulatory functions of cocaine may positively contribute to HIV-1 infection and replication. For example, cocaine has been proposed to negatively modulate the expression of HIV-suppressing chemokines and their receptors (Nair et al., 2000; Roth et al., 2005; Reynolds et al., 2009) in target cells. These include RANTES, MIP-1a and MIP-1b, which are all known to inhibit binding/fusion/entry of the virus (Nair et al., 2000, 2001). Furthermore, cocaine has been reported to upregulate the expression of the viral entry co-receptors CXCR4 and CCR5 (Nair et al., 2000, 2001), and, as a result, enhance HIV-1 replication by increasing viral entry. However, accumulating evidence indicate that cocaine enhances HIV-1 infection by targeting both entry and post-entry steps of HIV-1 life cycle (Nair et al., 2000, 2001; Mantri et al., 2012; Addai et al., 2015).

HIV-1 entry into the target cells is facilitated by the binding of viral glycoproteins (gp120 and gp41) to the CD4 receptor and the chemokine co-receptors (CCR5 and CXCR4; Telesnitsky and Goff, 1997). The ensuing fusion of the viral and cell membranes leads to the release of the viral capsid core into the cytoplasm of the target cells (Telesnitsky and Goff, 1997). The subsequent post-entry events of HIV-1 replication can be broadly categorized into reverse transcription, integration, transcription, translation, assembly, virus release and maturation (Telesnitsky and Goff, 1997). We and other research groups have reported that cocaine can modulate post-entry steps of HIV-1 life cycle (Mantri et al., 2012; Kim et al., 2013; Addai et al., 2015). Cocaine has been shown to downregulate the cellular miRNA “miR-125b” in CD4^+^ T cells (Mantri et al., 2012). The miR-125b is a member of the of anti-HIV miRNA family that suppresses HIV-1 replication by binding to the viral transcripts and inhibiting their translation (Huang et al., 2007). Cocaine was also recently shown to downregulate miR-155 that negatively regulates DC-SIGN (Napuri et al., 2013), which plays a critical role in HIV-1 infection of dendritic cells. A proteomic study of cocaine-treated human astrocytes revealed that cocaine differentially regulates an array of cellular proteins, some or many of which were postulated to potentially modulate HIV-1 replication (Reynolds et al., 2006). Cocaine’s potentiating effect on HIV-1 has been partly attributed to the drug-induced upregulation of sigma-1 receptor, an endoplasmic reticulum-resident molecular chaperone known to bind cocaine. Cocaine’s potentiating effect on HIV-1 has been partly attributed to the drug-induced upregulation of sigma-1 receptor, an endoplasmic reticulum-resident molecular chaperone known to bind cocaine (Ramamoorthy et al., 1995; Roth et al., 2005). More recently, another receptor for cocaine, dopamine D4 receptor (D4R), was shown to mediate the drug-induced enhancement of HIV-1 infection in quiescent CD4^+^ cells, as evidenced by increased reverse transcription kinetics (Kim et al., 2013). Recently, our group demonstrated that cocaine increases HIV-1 proviral integration in CD4^+^ T cells (Addai et al., 2015). Increased HIV-1 integration could enhance viral transcription and viral protein production. Cumulatively, these studies provide strong evidence that cocaine can modulate both entry and post-entry steps of HIV-1 infection, thus contributing to increased viral load.

#### Cocaine’s Effect on CD4^+^ T cell Decline

CD4^+^ T cell decline is a powerful diagnostic marker for HIV-1 disease progression (Embretson et al., 1993; Heinkelein et al., 1995; Lane, 2010). Studies suggest that cocaine abusing HIV patients have lower CD4^+^ T cell counts and accelerated decline in CD4^+^ T cell number (Chaisson et al., 1989; Anthony et al., 1991; Ruiz et al., 1994; Friedman et al., 2006; Cook, 2011). Compared to non-addicts, individuals addicted to cocaine had significantly reduced T cell population (Ruiz et al., 1994). Further supportive evidence comes from animal studies demonstrating that cocaine decreases the number of circulating lymphocytes (Evans et al., 1996; Pellegrino and Bayer, 1998; Colombo et al., 1999). There is also evidence that cocaine modulates the proliferation of human T cells (Klein et al., 1993; Matsui et al., 1993). Results from *in vitro* experiments by our laboratory indicate that when CD4^+^ T cells were treated with both cocaine and HIV-1 virions, the extent of CD4^+^ T cell apoptosis is significantly higher compared to that of cells treated with cocaine or HIV-1 virions alone (Pandhare et al., 2014). This suggests that, albeit in cell culture system, cocaine and HIV-1 act in a synergistic fashion in inducing CD4^+^ T cell death (Pandhare et al., 2014). While the precise mechanism by which cocaine synergizes with HIV-1 in the CD4^+^ T cell decline remains unclear, work done in our laboratory suggest that increase in reactive oxygen species (ROS) may play an important role (Pandhare et al., 2014). Furthermore, it was shown that cocaine downregulates the anti-apoptotic microRNA “miR-125b” in uninfected and infected CD4^+^ T cells (Mantri et al., 2012). miR-125b is a known negative regulator of the pro-apoptotic gene p53 (Le et al., 2009, 2011), and hence the cocaine-induced downregulation of this miRNA could potentially play an important role in the underlying synergy between cocaine and HIV-1 in CD4^+^ T cell death. More recently, we also reported that cocaine directly enhances HIV-1 integration in CD4^+^ T cells (Addai et al., 2015). This conceivably could account for the deleterious effects of cocaine abuse on accelerated CD4^+^ T cell apoptosis in HIV-1 patients, because increased viral integration has been proposed to induce CD4^+^ T cell apoptosis (Cooper et al., 2013). Given that both viral load and CD4^+^ T cell death are important predictors of HIV-1 disease progression, findings from our laboratory and others highlight the potentiating effects of cocaine on HIV-1 disease progression in drug abusing patients.

### HIV-1 Infection in the Brain

HIV-1, besides its well-characterized negative effects on the host immune system through depletion of infected CD4^+^ lymphocytes and immune activation, also broadly impacts the nervous system (Gonzalez-Scarano and Martin-Garcia, 2005; Spudich and Gonzalez-Scarano, 2012). During the early phase of infection, HIV-1 enters the brain and persists in the CNS (Gonzalez-Scarano and Martin-Garcia, 2005; Spudich and Gonzalez-Scarano, 2012). Macrophages and microglia are the primary cell types in the central CNS that can be infected by HIV-1 as well as support viral replication (Koppensteiner et al., 2012). Although neurons are refractory to HIV-1 infection, progressive neuronal damage has been observed in HIV-1 infected patients (Berger and Nath, 1997; Clifford, 2000; Kaul et al., 2001; McArthur et al., 2010). HIV-1 infected individuals suffer from a wide range of neurological disorders. The invasion of the brain by the, leads to a battery of deficits including reduction in motor speed, decline in memory and concentration, CNS injury and neuropathy, which are collectively termed as HIV-associated neurocognitive disorders (HAND; Woods et al., 2009; Patel et al., 2013; Sanmarti et al., 2014). From a clinical perspective, HAND is strongly linked to neurological disorders namely asymptomatic neurocognitive impairment (ANI), mild neurocognitive disorder (MND), and HIV- associated Dementia (HAD)- the most severe form of HAND (Woods et al., 2009; Patel et al., 2013; Sanmarti et al., 2014). The introduction and the widespread use of ART has managed to keep the prevalence of HAD in check. However, the incidences of other milder forms of HAND still persist, are widespread, and are increasing globally (Gray et al., 2003; Tozzi et al., 2007; Joska et al., 2010). HAND affects 20–30% patients in the late stages of AIDS and is believed to be the most common cause of dementia among people aged 40 or less worldwide (Albright et al., 2003; Vivithanaporn et al., 2010). Cognitive, motor, and behavioral dysfunctions are the key phenotypes accompanying neuronal cell injury and death that result from HIV-1 infection of the nervous system in individuals across various age groups (Woods et al., 2009; Patel et al., 2013; Sanmarti et al., 2014). In adult patients, the virus-induced disruption of the nervous system includes targeted damage to regions beneath the cortex, which significantly depletes basal ganglia levels (Aylward et al., 1993; Berger and Nath, 1997). Pathological features of HAND are characterized by monocyte infiltration into brain, formation of macrophage-derived multinucleated giant cells, microglial nodules, gliosis, myelin pallor, and neuronal loss (Gelman, 2015). Both a person’s age and length of HIV-1 infection are key factors for developing HAND because prolonged infection accompanied by a decline in CD4^+^ T cell count indicates an increased persistence of the virus in the brain (Tozzi et al., 2007; Brew and Letendre, 2008). Further, co-morbidities such as hepatitis C virus (HCV) infection and abuse of drugs such as cocaine also increase the incidence of HAND (Gill and Kolson, 2014; Tedaldi et al., 2015).

#### Blood–Brain Barrier

**FIGURE 1:**
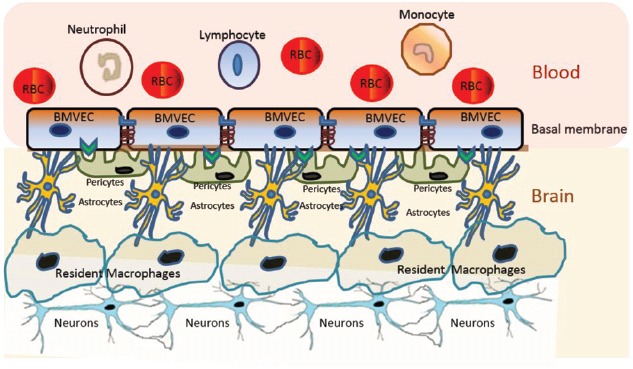
**Schematic representation of the BBB.** The BBB is made up of a continuous layer of tightly linked BMVECs. The BMVECs are supported by the basal lamina, followed by the brain parenchyma that consists of astrocytes, pericytes, perivascular macrophages, microglia, and neurons. Each component of BBB plays critical roles in maintaining the selective permeability of molecules for neuronal homeostasis and protecting the brain from pathogens and xenobiotics.

The blood–brain barrier (BBB) is a very dynamic structure critical for maintaining brain homeostasis and protecting the brain from pathogens and xenobiotics (Ballabh et al., 2004). The BBB separates the CNS from the periphery and acts as the interface between blood capillaries and the brain parenchyma (Ballabh et al., 2004; Figure 1). The BBB is made up of a continuous layer of tightly linked brain microvascular endothelial cells (BMVECs) that is supported by the basal lamina, followed by the brain parenchyma that consists of astrocytes, pericytes, perivascular macrophages, microglia, and neurons (Ballabh et al., 2004). Pericytes are cells of connective tissue that sustain the BBB by regulating its permeability, capillary blood flow, and phagocytosis of cellular debris (Ivey et al., 2009). The BMVECs, in combination with tight junction proteins, form an unperforated endothelium that confers BBB the selective permeability to peripheral proteins and other external molecules (Stamatovic et al., 2008; Lv et al., 2010). Specifically, BMVECs maintain selective permeability via the luminal membrane-resident transport systems, which modulate the efflux and influx of biomolecules through the BBB (Stamatovic et al., 2008; Lv et al., 2010).

#### Alterations in the Brain and the BBB by HIV-1 Infection

The CNS is a major target for HIV-1 infection and, because many antiretroviral drugs display high variability in penetrating the BBB and reaching therapeutic concentrations, also serves as a reservoir for the virus (Ivey et al., 2009; Stamatovic et al., 2008; Lv et al., 2010). The precise mechanism by which HIV-1 crosses the BBB and causes neuronal dysfunction remains poorly understood. Findings from animal models and *in vitro* studies provide evidence supporting the “Trojan Horse” model for HIV entry into the brain (Gonzalez-Scarano and Martin-Garcia, 2005; Ivey et al., 2009; Hazleton et al., 2010). This model suggests that HIV-1 virions cross the BBB via trafficking of infected CD4^+^ cells and monocytes into the CNS (Gonzalez-Scarano and Martin-Garcia, 2005; Ivey et al., 2009; Hazleton et al., 2010). Accordingly, infected monocytes cross the BBB more efficiently than uninfected monocytes (Persidsky, 1999; Persidsky et al., 1999). Further, prevalence of increased numbers of non-proliferating monocytes and brain macrophages in the CNS of HIV-infected patients is observed as a clinical outcome (Fischer-Smith et al., 2004). Infected monocytes that have traversed into the CNS do possess the ability to repopulate as the resident macrophages (Koppensteiner et al., 2012). Despite some evidence pointing to HIV-1 entering the brain by direct migration or transcytosis of endothelial cells through the BBB, the current prevailing model of CNS infection focuses on infected circulating monocytes as viral cargos crossing the BBB into the CNS throughout infection. Once in the brain, HIV-1 can infect perivascular macrophages and microglia, both of which express the CD4^+^ receptor. Astrocytes can be infected but are not a site of active HIV replication. Neurons do not express the CD4^+^ receptor and thus are not capable of productive infection; therefore, the HIV-associated neurological complications causing neuronal damage mainly result from inflammatory factors and neurotoxic substances released by infected as well as activated, uninfected cells.

**FIGURE 2:**
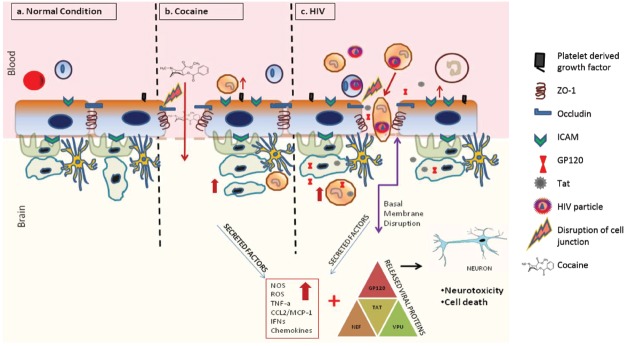
**Effects of cocaine and HIV-1 on BBB integrity and neuronal toxicity.** Cocaine and HIV-1 infection has been known to disrupt the integrity of BBB. This breach in BBB can increase trafficking of infected cells, viral particles and/or viral proteins into the brain causing systemic neuro-inflammation. Ongoing neuro-inflammation and low level of HIV-1 replication causes neuronal dysfunction and neurotoxicity. Furthermore, drugs of abuse such as cocaine can synergistically accentuate HIV associated neuronal dysfunction and neurotoxicity leading to worsening of HIV-1 neuropathogenesis.

One of the hallmarks of HAND pathogenesis is the breach of the BBB by infected cells. HIV-1 infection has been shown to compromise the BBB integrity (Atluri et al., 2015; Figure 2). Specifically, multiple studies suggest that several HIV-1 proteins can regulate the permeability of the BBB (Toborek et al., 2005). For example, administration of the HIV-1 Trans Activator (Tat) protein, which traverses the BBB potentially by adsorptive endocytosis (Banks, 2005), in mice led to decreased expression of occludin and ZO-1 proteins in the tight junction of BBB (Pu et al., 2007). Further analysis revealed activation of cyclooxygenase 2 (Cox-2) as the mechanistic basis behind the diminished expression of these tight junction proteins (Pu et al., 2007). *In vitro* treatment of various cell types, including BMVEC’S, with Tat led to increased oxidative stress, which could be attributed to disruption of tight junctions of the BBB (Shiu et al., 2007). In astrocytes, Tat reportedly elevates the expression levels of matrix metalloproteinase-9 (MMP-9; Ju et al., 2009), a member of the zinc-dependent endopeptidases known to cleave the extracellular matrix proteins. Accordingly, MMPs such as MMP-9 have been reported to disrupt the BBB by degrading the extracellular matrix proteins such as laminin and type IV collagen (Yong et al., 1998). Moreover, Tat has been shown to act as a potential chemo attractant in the BBB milieu, which potentially could alter BBB integrity. There is also some evidence that Tat acts as a potential chemo attractant in the BBB milieu that may alter BBB integrity.

The HIV-1 gp120, which mediates viral entry by binding the CD4 receptor and CXCR4 or CCR5 co-receptor on host cell surface, has also been reported to breach the BBB. For instance, treatment of cultured human BMVECs with gp120 increased monocyte migration and caused a dose-dependent decrease in transendothelial electrical resistance (TEER; Kanmogne et al., 2007). These alterations were postulated to be consequences of increased permeability of BBB caused by the activation of myosin light chain kinase and/or protein kinase C and increased Ca^2+^ flux (Kanmogne et al., 2007). Furthermore, *in vitro* treatment of human BMVEC’s with gp120 also decreased BBB tight junction proteins such as ZO-1, ZO-2 and occludin (Kanmogne et al., 2005; Nakamuta et al., 2008). Exposure of THP-1 monocytes to gp120 has also been shown to increase secretion of neurotoxins (Pietraforte et al., 1994). Cytokines could also play a major role in the gp120-induced alteration of the integrity of BBB, as evidenced from studies reporting enhanced expression of pro-inflammatory cytokines due to gp120 treatment *in vitro* (Ronaldson and Bendayan, 2006). Besides Tat and gp120, other HIV-1 proteins have also been implicated in modulating BBB function (Albright et al., 2003).

### Cocaine Abuse and HIV-1 Neuropathogenesis

The complexity of HAND is further exacerbated by co-morbid conditions such as drug abuse. Cocaine has been suggested to increase HAND pathogenesis which continues to be a major challenge in the post ART era (Pakesch et al., 1992; Larrat and Zierler, 1993; Goodwin et al., 1996; Nath et al., 2001; Ferris et al., 2008; Norman et al., 2009). HIV-positive cocaine users are cognitively more impaired and poorly adhere to ART than their respective non-drug-abusing counterparts (Meade et al., 2011). Basal ganglia damage that may lead to basal ganglia dysfunction has been reported in HIV-infected cocaine users (Newsome et al., 2011). Cocaine is also known to regulate the rapid activation of the hypothalamic-pituitary-adrenal axis due to enhanced norepinephrine activity (Torres and Rivier, 1992; Sarnyai et al., 1996; Manetti et al., 2014). Neurodegeneration and neuro inflammation are the hallmark features of HAND (Gill and Kolson, 2014; Rao et al., 2014), and are often exacerbated in the presence of drugs of abuse such as cocaine (Gannon et al., 2011; Buch et al., 2012). Brain autopsy studies revealed significant prevalence of microglia activation, BBB disruption and infiltration of multinucleated giant cells in drug-abusing HIV-positive individuals over non-abusing HIV-positive controls (Gannon et al., 2011). Besides its positive effect on HIV-1 replication in macrophages (Dhillon et al., 2007) and microglia (Gekker et al., 2006), cocaine use has also been shown to enhance HIV-1 replication in the normal human astrocytes (NHAs; Reynolds et al., 2006). Cocaine-exposed, HIV-infected NHAs exhibited upregulated p24 antigen levels, indicative of high viral protein content (Reynolds et al., 2006). Though neurons are generally refractory to HIV-1, persistent and low level viral infection in the brain triggers production of neurotoxic factors and release of proinflammatory cytokines and chemokines from HIV-1 infected macrophages/microglia as well as astrocytes, thus causing neuroinflammation and neurotoxicity that culminates in HAND (Gonzalez-Scarano and Martin-Garcia, 2005; Rao et al., 2014). The plasma and post-mortem brain tissue of HIV-1 infected cocaine users, when compared to that of non-drug users, displayed elevated levels of the lysosomal protease cathepsin B, a neuronal pro-apoptotic factor secreted by activated macrophages (Zenon et al., 2014). This response could be recapitulated when HIV-infected MDM were exposed to cocaine, thereby demonstrating that cocaine potentiates cathepsin B secretion and increases neuronal apoptosis (Zenon et al., 2014). Cocaine abuse also increases expression of acute phase proteins such as C-reactive protein (CRP) and serum amyloid A (SAA), which cause chronic inflammatory effects, including in the brain (Samikkannu et al., 2013). Thus, cocaine abuse not only facilitates HIV-1 neuro-invasion via its effect on the BBB but also enhances neuroinflammation and neurotoxicity observed in HIV-1 infected individuals. In summary, the interplay between cocaine and HIV-1 in the CNS mediated via a myriad of pathways, causes deleterious effects leading to worsening of HAND.

#### Cocaine Alters Integrity of the BBB

Cocaine, besides being able to passively diffuse through the BBB, has also been shown to breach the integrity of the BBB by modulating the structural components of the barrier endothelium (Sharma et al., 2009). Though the biochemical mechanism behind this effect awaits delineation, cocaine’s ability to alter the expression of proteins such as intracellular adhesion molecule 1 (ICAM-1), vascular cell adhesion molecule 1 (VCAM-1), and endothelial-leukocyte adhesion molecule (ELAM or selectin-1) has been postulated as a key contributory factor (Fiala et al., 1998, 2005; Kousik et al., 2012). This has been shown, both *in vitro* and in rodents, to trigger increased leukocyte migration across endothelial monolayers, accompanied by elevated levels of pro-inflammatory cytokines and chemokines such as tumor necrosis factor alpha (TNF-*α*), nuclear factor kappa B (NFkB), interleukin 6 (IL-6), and others, ultimately resulting in neuro-inflammation (Fiala et al., 1998; Chang et al., 2000). In addition, cocaine up-regulates the pro-migratory CCL2/CCR2 ligand-receptor system, thereby enabling the HIV-infected monocytes cross the BBB (Dhillon et al., 2008). *In vitro*, cocaine binds to its cognate receptor σ-1-R in BMVECs and induces the expression of platelet-derived growth factor (PDGF), both at the transcript and protein levels, thereby enhancing endothelial permeability (Yao et al., 2011). Importantly, treatment with PDGF neutralizing antibodies abrogated this effect in mice (Hata et al., 2010). Cocaine exposure also leads to BBB deregulation via the loss or modulation of tight junction proteins. For example, cocaine downregulates the expression of the tight junction protein ZO-1 (Dhillon et al., 2008). Furthermore, cocaine is also known to upregulate matrix metalloproteinase (MMP) expression which triggers reorganization of the basement membrane (Nair et al., 2005). The ensuing damage to the integrity of the BBB has been suggested to facilitate the entry of the virus into the brain. Thus, cocaine-induced alterations in the BBB integrity can be hypothesized to potentiate HAND pathogenesis by enhancing HIV-1 neuroinvasion, an outcome facilitated by increased monocyte entry and neuroinflammation.

#### Cocaine Exacerbates the Toxicity of HIV-1 Proteins in the Brain

Cocaine, has been reported to amplify the neurotoxic effects of HIV-1 proteins such as Tat and gp120 released from the infected cells, with a magnitude that exceeds that of cocaine or HIV alone. Both *in vitro* and *in vivo* studies have shown that mitochondrial dysfunction and oxidative stress leading to neuronal cell damage and death are key factors in exacerbating the effects of cocaine on the pathophysiology of neurodegenerative disorders. Treatment of primary rat hippocampal neurons with Tat protein in the presence of cocaine induced a synergistic effect that enhanced neurotoxicity (Aksenov et al., 2006). Cocaine also enhanced the Tat-induced mitochondrial depolarization and generation of intracellular ROS; Aksenov et al. (2006). Pre-treatment with a specific dopamine receptor antagonist blocked the cocaine-induced potentiation of Tat neurotoxicity, thereby underpinning the role of dopamine receptor-mediated signaling (Aksenov et al., 2006). Likewise, combinatorial toxicity studies have demonstrated that exposure of rat primary neurons to both cocaine and gp120 led to increased oxidative stress that was accompanied by an increase in pro-apoptotic markers (Yao et al., 2009). In another *in vivo* study, administering rats with a combination of cocaine and ventricular injection of HIV-1 gp120 protein resulted in increased iNOS (nitric oxide synthase) levels accompanied by neuronal apoptosis in the neocortex region (Bagetta et al., 2004). Importantly, this effect was ameliorated in animals pretreated with the iNOS inhibitor, thus substantiating the involvement of iNOS in HIV-1 gp120 and cocaine-mediated neuronal apoptosis (Bagetta et al., 2004). Furthermore, treatment of hippocampal neurons with both HIV-1 gp120 and cocaine resulted in enhanced loss of neuronal dendrites (Bagetta et al., 2004). Together, these studies demonstrate that cocaine augments the effect of HIV-1 Tat and gp120 on neurotoxicity that may contribute to enhanced HIV-associated neuropathogenesis.

#### Cocaine Elicits Immuno-modulatory Effects on Brain

Cocaine has multiple physiologic and pathological effects on various organ systems including the CNS. In general, CNS comprises of the brain and the spinal cord. Brain is composed of two major cell types (Glial cells and Neurons) contributing to its conserved uniqueness in terms of memory, learning and motor activity. Neurons carry and transmit signals across specialized synaptic junctions, whereas glial cells maintain the structural and metabolic homeostasis of the CNS. There is evidence that cocaine disrupts the homeostasis in the CNS by exerting toxic and immunomodulatory effects. Persistent inflammation in the brain due to cocaine abuse has been shown to involve neuroendocrine interactions. For example, cocaine modulates secretion of pituitary-adrenocortical hormones that are immunomodulatory (Rivier and Vale, 1987; Mendelson et al., 1992). Cocaine can also directly modulate cellular immune responses by either interacting with specific membrane-associated binding sites or by non-specific interaction with the lymphocyte plasma membrane (Van Dyke et al., 1976; Klein et al., 1993; Matsui et al., 1993). Cocaine’s addictive effects are dependent on the blockage of the DAT and intensification of dopamine neurotransmission (Ritz et al., 1987; Kuhar, 1992; Woolverton and Johnson, 1992). Furthermore, dopamine treatment increased entry of HIV-1 virions into macrophages invoking the possibility that CNS dopamine may be a common mechanism by which cocaine may exacerbate neuroinflammation (Gaskill et al., 2014). In addition to the dopamine system, sigma receptors are also involved in cocaine mediated alterations in immune and neuronal function (Maurice et al., 2002). The sigma1 (σ1) receptor, in particular, has been associated with altered immune functionality because it is expressed in many cells in the CNS and PBMCs (Wolfe et al., 1989; Maurice et al., 2002).

Despite advances in understanding the addictive effects of cocaine, the molecular details behind the immunomodulatory effects of cocaine in the brain are unclear. Cocaine has been shown to modulate immune cell activity and secretion of pro-inflammatory cytokines and chemokines, which together lead to hypersensitive and perturbed immune responses (Baldwin et al., 1998; Pellegrino and Bayer, 1998; Clark et al., 2013). Cocaine users reportedly harbor significantly high numbers of activated microglia and macrophages (Bowers and Kalivas, 2003; Little et al., 2009), and recent evidence indicates that cocaine can activate innate immune signaling within the brain. The innate immune system in brain is comprised primarily of microglial cells, and cocaine has been shown to activate the pattern-recognition receptor Toll-like receptor 4 (TLR4) present on the microglial cells, thereby causing the release of proinflammatory cytokine interleukin-1β (IL-1β; Northcutt et al., 2015). These proinflammatory signals contribute to cocaine-induced changes in the mesolimbic dopamine system and cocaine reward (Cearley et al., 2011). In addition, cocaine has been shown to activate astrocytes directly or indirectly through the microglial proinflammatory response (Bowers and Kalivas, 2003; Coller and Hutchinson, 2012). Upon activation, astrocytes also release proinflammatory cytokines and glutamate, thus altering the glutamatergic signaling and neuronal excitability (Kalivas and Hu, 2006). Chronic cocaine use can cause sustained activation of microglia and astrocytes, and the ensuing excessive extracellular glutamate can cause neurotoxicity via glutamatergic dysregulation. In summary, the immune-modulatory effect of cocaine can elicit neurotoxic and addictive effects in the brain that contribute to enhanced HIV-1 induced neuropathogenesis.

### Conclusions and Future Prospective

An in-depth understanding of the synergy between HIV-1 and drugs of abuse such as cocaine is essential to address and effectively combat the HIV/AIDS global pandemic. Even as ART has significantly reduced AIDS-related mortalities, averting the neuro-pathological effects of HIV-1 infection in the brain remains a daunting challenge. This is all the more urgent for drug users, as accumulating evidence suggest that drug use accentuates HIV-1 neuropathogenesis. One major contributor for this exacerbation is the low-level persistent viral replication in the brain that drives neuroinflammation. This complex interplay between the virus and the interfering drugs is further aggravated by the latter’s inherent excitotoxic potential in targeting the CNS at cellular and genetic level. Strategies focused on priority areas of action are indeed required to bolster native brain defense mechanisms toward nullifying the effects of drugs of abuse on HIV-1 pathogenesis. However, our limited mechanistic understanding highlights the need of systematic *in vivo* model systems to elucidate the cellular circuitries involved in cocaine-mediated HIV-1 toxicity. On that note, an in-depth analysis of potential roles of miRNAs in this complex nexus between the virus and cocaine may yield valuable insights. In addition, whether cocaine interacts with the wild type and drug resistant HIV-1 differently that may accentuate disease progression is yet to be determined. Together, identification of pathways and regulatory molecules behind the effect of cocaine on HIV-1 pathogenesis could greatly assist in target selection for therapeutic intervention, in the days to come.

### Conflict of Interest Statement

The authors declare that the research was conducted in the absence of any commercial or financial relationships that could be construed as a potential conflict of interest.

## Footnotes

1 http://www.who.int/mediacentre/factsheets/fs360/en/index.html

2 http://nsduhweb.rti.org/respweb/homepage.cfm
